# Feasibility of Optical Coherence Tomography (OCT) for Intra-Operative Detection of Blood Flow during Gastric Tube Reconstruction

**DOI:** 10.3390/s18051331

**Published:** 2018-04-25

**Authors:** Sanne M. Jansen, Mitra Almasian, Leah S. Wilk, Daniel M. de Bruin, Mark I. van Berge Henegouwen, Simon D. Strackee, Paul R. Bloemen, Sybren L. Meijer, Suzanne S. Gisbertz, Ton G. van Leeuwen

**Affiliations:** 1 Department of Biomedical Engineering & Physics, Academic Medical Center, University of Amsterdam, 1105 AZ Amsterdam, The Netherlands; m.almasian@amc.uva.nl (M.A.); l.s.wilk@amc.uva.nl (L.S.W.); d.m.debruin@amc.uva.nl (D.M.d.B.); p.r.bloemen@amc.uva.nl (P.R.B.); t.g.vanleeuwen@amc.uva.nl (T.G.v.L.); 2 Department of Plastic, Reconstructive & Hand Surgery, Academic Medical Center, University of Amsterdam, 1105 AZ Amsterdam, The Netherlands; s.d.strackee@amc.uva.nl; 3 Department of Surgery, Academic Medical Center, University of Amsterdam, 1105 AZ Amsterdam, The Netherlands; m.i.vanbergehenegouwen@amc.uva.nl (M.I.v.B.H.); s.s.gisbertz@amc.uva.nl (S.S.G.); 4 Department of Pathology, Academic Medical Center, University of Amsterdam, 1105 AZ Amsterdam, The Netherlands; s.l.meijer@amc.uva.nl

**Keywords:** flow, monitoring, OCT, optical imaging, surgery, esophagectomy, gastric tube, perfusion, speckle

## Abstract

In this study; an OCT-based intra-operative imaging method for blood flow detection during esophagectomy with gastric tube reconstruction is investigated. Change in perfusion of the gastric tube tissue can lead to ischemia; with a high morbidity and mortality as a result. Anastomotic leakage (incidence 5–20%) is one of the most severe complications after esophagectomy with gastric tube reconstruction. Optical imaging techniques provide for minimal-invasive and real-time visualization tools that can be used in intraoperative settings. By implementing an optical technique for blood flow detection during surgery; perfusion can be imaged and quantified and; if needed; perfusion can be improved by either a surgical intervention or the administration of medication. The feasibility of imaging gastric microcirculation in vivo using optical coherence tomography (OCT) during surgery of patients with esophageal cancer by visualizing blood flow based on the speckle contrast from M-mode OCT images is studied. The percentage of pixels exhibiting a speckle contrast value indicative of flow was quantified to serve as an objective parameter to assess blood flow at 4 locations on the reconstructed gastric tube. Here; it was shown that OCT can be used for direct blood flow imaging during surgery and may therefore aid in improving surgical outcomes for patients.

## 1. Introduction

The viability of cells and tissue mainly depends on blood flow as it transports oxygen and nutrients to the cells. Without oxygen and nutrients, ischemia occurs and tissue becomes necrotic [1]. An esophageal resection with ensuing gastric tube reconstruction is the cornerstone of treatment in patients with esophageal cancer. To be able to pull up the gastric tube, ligation of the left gastric artery, the left gastro-epiploic artery, the short gastric vessels and some branches of the right gastric artery is needed. As a result, perfusion of the tube’s gastric tissue after reconstruction relies on the right gastroepiploic artery and some branches of the right gastric artery [2] leaving the future neo-esophageal anastomotic site depending only on collateral blood flow.

Anastomotic leakage (incidence 5–20%) and stricture (10–22%) are major complications following esophagectomy, and mortality is as high as 4% [2]. Perfusion deficiency of the gastric tube is seen as the major risk factor to develop these complications, which, in turn, correlate with high morbidity, IC-unit stay, high costs in healthcare and decreased quality of life [3]. Monitoring perfusion would allow surgeons to make different choices in their surgical design [4] and, if needed, involve anesthesiology interventions to optimize perfusion by the use of fluid or medication [5]. Consequently, intra-operative perfusion monitoring could potentially aid in achieving better patient outcomes after surgery and in decreasing complications and mortality. 

Optical techniques are well-suited for intra-operative monitoring due to their minimal-invasive and real-time visualization capabilities [6]. Optical Coherence Tomography (OCT) allows high-resolution, non-invasive, real-time imaging of tissue [7]. It detects backscattered near-infrared light from tissue to obtain depth-resolved, in vivo images. As a result, this technique potentially allows visualization of vasculature in different tissue layers until a depth of approximately 2.5 mm [8]. Moreover, the underlying arteries will not influence the measured perfusion in the overlaying microvascular network, in contrast to other optical imaging techniques like fluorescence imaging, laser speckle contrast imaging or laser Doppler flowmetry [9,10,11]. Finally, a handheld OCT probe is easy to use in the operation room. 

A large number of studies have investigated and established the link between OCT speckle and the flow of the imaged medium, ranging from qualitative detection of flow to quantitative analysis of the flow parameter in very controlled measurement settings [8,12,13,14]. By analyzing the speckle variance [15] or speckle decorrelation [12] of OCT data, flow can be discriminated from static tissue in order to visualize blood vessels and microcirculation. In previous studies we have shown that OCT speckle decorrelation can be used to obtain a quantitative blood flow parameter [8,14]. However, because of the needed fixation during imaging, these systems are not readily applicable in the operating room. Therefore, for the visualization of flow during surgery, in this study we use speckle contrast in the OCT M-mode scans to distinguish flow from static tissue.

The aim of this study is to research the feasibility of a commercially available OCT system to detect blood vessels in gastric tissue, in patients with esophageal cancer in a clinical setting during esophageal cancer surgery with gastric tube reconstruction. 3D OCT scans are obtained to image tissue layers in the reconstructed gastric tube and compared to histopathological slides, yielding information about tissue structure and blood vessel locations. In this paper, speckle contrast, the ratio of speckle variance over the mean, is calculated from OCT M-mode scans to distinguish areas with flow from static tissue. The percentage of pixels indicative of flow is used as an objective parameter to compare blood flow at different locations, ranging from normal perfusion, near the remaining right gastroepiploic artery and/or branches of the right gastric artery, to decreased perfusion, near the future anastomotic side.

## 2. Materials and Methods

This prospective, observational, in vivo pilot study of 26 patients with esophageal cancer who underwent an esophageal resection with gastric tube reconstruction was approved by the medical ethics committee (NL52377.018.15) of the Academic Medical Center of Amsterdam, and submitted at the clinicaltrials.gov database (NCT02902549) [16]. Patients were included in this study between October 2015 and June 2016 in the Academic Medical Center (Amsterdam, The Netherlands). Written informed consent was obtained at least one week before surgery. Surgery was performed by two experienced upper-gastrointestinal surgeons (MIvBH, SSG).

**Figure 1:**
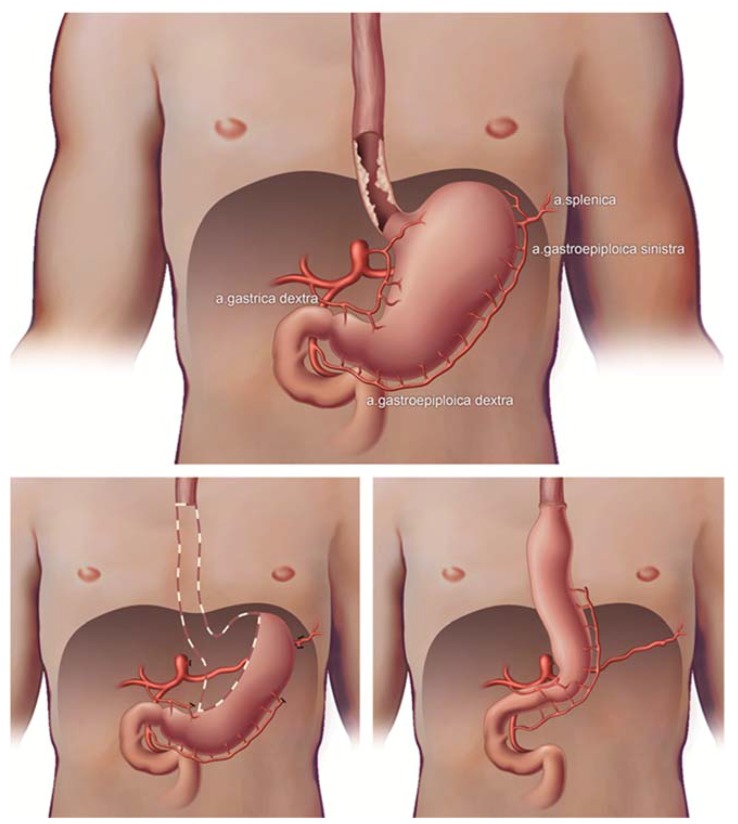
Esophageal cancer with gastric vascularization, esophagectomy and gastric tube reconstruction with only one artery left (gastroepiploic artery).

Usually, patients undergo a minimally invasive Ivor Lewis procedure (2-stage procedure with intra-thoracic anastomosis), however, in case of a mid- or proximal esophageal carcinoma, a minimally invasive McKeown procedure (3-stage procedure with cervical anastomosis) was performed. Mobilization and vascularization of the gastric conduit was the same in both procedures. The procedures have been described in detail before [5]. In brief, during the abdominal phase a lymphadenectomy and mobilization of the stomach was performed, ligating the left gastric artery, some branches of the right gastric artery at the level of the angulus, the left gastro-epiploic artery and the short gastric vessels (Figure 1). During the thoracic phase a lymphadenectomy was performed, the esophagus was mobilized and after extraction of the specimen and gastric pull-up an intrathoracic or a cervical anastomosis was created. In all patients, a 3–4 cm wide gastric tube was reconstructed using a powered ECHOLON FLEX Stapler (Ethicon, Johnson & Johnson Health Care Systems, Piscataway, NJ, USA). Branches of the right gastric artery supply the remains of the lesser curvature. The right gastro-epiploic artery supplies the greater curvature until the watershed area with the left gastro-epiploic artery.

**Figure 2:**
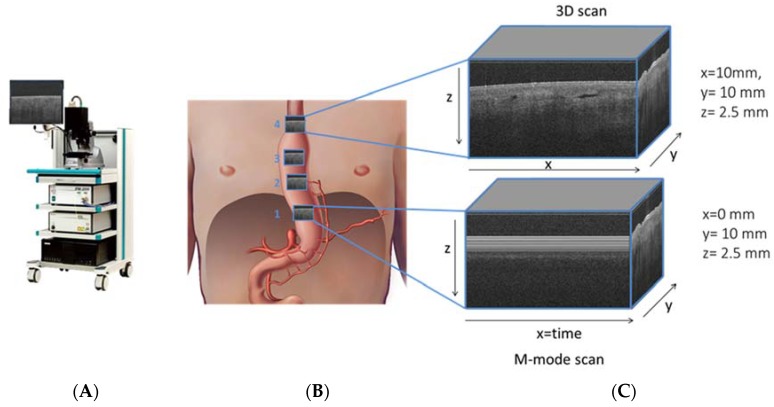
Santec OCT system (panel **A**), schematic figure (panel **B**) of gastric tube with ROI of 10 × 10 mm of OCT grayscale images at four perfusion areas, with shadowing of vessels in cross sectional OCT image (panel **C**).

OCT data were recorded using a commercial 50 kHz IVS 2000 swept source OCT system (THORLABS, Newton, NJ, USA) operating at a center wavelength of ~1300 nm. The full width half maximum axial and lateral resolutions were measured to be ~14 μm and ~25 μm, respectively. Volumetric images (x, y, z) of 10 mm by 10 mm by 2.5 mm, containing 1024 by 1024 by 400 pixels, and M-mode scans (x, y, z) of 0 mm by 10 mm by 2.5 mm, containing 1024, 1024, 400 pixels were collected. The depth axis was corrected for the refractive index of tissue (n_ref_ = 1.4) (Figure 2).

A sterile sheet was placed around the probe for the intra-operative measurements on gastric tissue. Measurements were taken with a hand-held OCT probe directly after preparation of the gastric tube, at four perfusion areas: 3 cm proximal of the level of the watershed between the right and left gastro-epiploic arteries (location 1), at the level of the watershed between the right and left gastro-epiploic arteries (location 2), 3 cm distal to the watershed between the right and left gastro-epiploic arteries (location 3) and at the level of the gastric fundus (location 4), physiologically from normal perfusion to decreased perfusion. Time to obtain data was recorded in the CRF.

Tissue from the fundus of the gastric tube was obtained for histology (*n* = 5) in 5 patients during surgery, with the aim to correlate these findings to the OCT scans. After routine processing of the tissue HE-stained, slides were digitized and evaluated by a pathologist (SLM) to define tissue layers and localize different structures, such as blood and lymph vessels. Different tissue layers (serosa, subserosa and muscularis propria) and blood and lymph vessels were annotated and compared to OCT scans.

**Figure 3:**
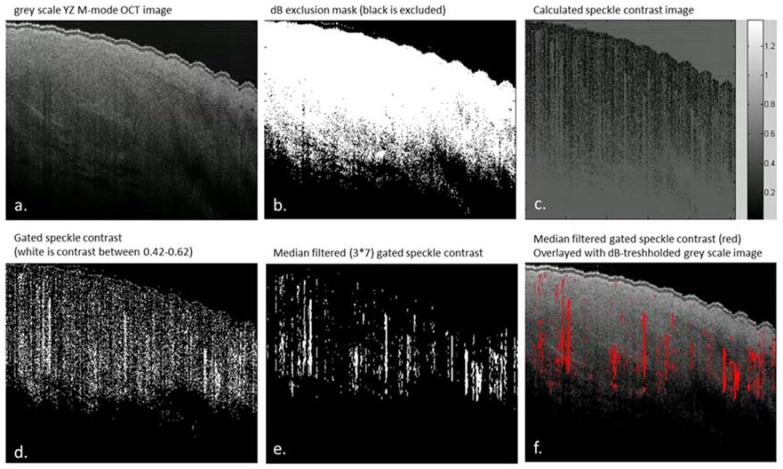
Data analysis steps of OCT M-mode scans, all images are shown from the y-z plane. (**a**) grayscale y-z M-mode scan; (**b**) applied dB mask to exclude noise from the data, the white areas on this image are included as data (**c**) over the time scale calculated speckle contrast values (of regions of included data after the dB threshold (**d**) speckle contrast within the 0.42–0.62 gate plotted in white (**e**) speckle contrast after applying a median filter with a 7 × 3 pixel kernel (**f**) gated and filtered speckle contrast (red) overlaid with the grayscale OCT image.

All data analysis was performed using custom-made scripts written in MATLAB (Mathworks, Natick, MA, USA). Using the M-mode scans, the speckle contrast (C) as a function of time was quantified in order to differentiate regions of flow from regions of static tissue. To this end, the following processing steps were applied to the data (Figure 3). First, a dB mask (−2 dB) was applied to exclude noise from the OCT data, where after a region of interest (ROI) in the Y-Z image is chosen to exclude corrupted parts of the scan, if needed. Second, the pixel-specific speckle contrast is calculated along the time axis for all pixels selected. The speckle contrast is the ratio of the amplitude variance over the mean amplitude [17]. The amplitude of regions with flow on OCT M-mode scans are expected to be Rayleigh distributed and hence have a speckle contrast value of 0.52 [18]. Here, we have used a speckle contrast gate between 0.42 and 0.62 to detect areas with flow. Next, a median filter with a kernel of 7 by 3 pixels is applied to the speckle contrast images. The pixels remaining after filtering are labeled as flow. The boundaries of the speckle contrast gate and the size of the median filter kernel are optimized by comparing the resulting speckle contrast images with the original X-Z M-mode scans by eye in five randomly chosen scans. Finally, an overlay is created using a Y-Z plot of the gated and filtered speckle contrast and the Y-Z grayscale OCT image. The percentage of pixels labelled as flow relative to the total number of pixels is calculated as an objective parameter to indicate the amount of vessels in the analyzed area.

The ability of the proposed method to distinguish pixels with flow from static tissue based on a speckle contrast gate between 0.42 and 0.62 was validated on a tissue mimicking flow phantom [8]. To simulate human perfusion heparinized human whole blood was flown with a velocity of 5 mm/s through a channel with a 100 µm diameter embedded in scattering silicon. The details on the manufacturing of the flow phantom are described elsewhere [8]. Single M-mode OCT scans (400 × 400 pixels) were collected at a static and flow region of the phantom and the speckle contrast was calculated as a function of depth.

## 3. Results

### 3.1. Patients and Feasibility of Intra-Operative OCT Imaging

**Figure 4:**
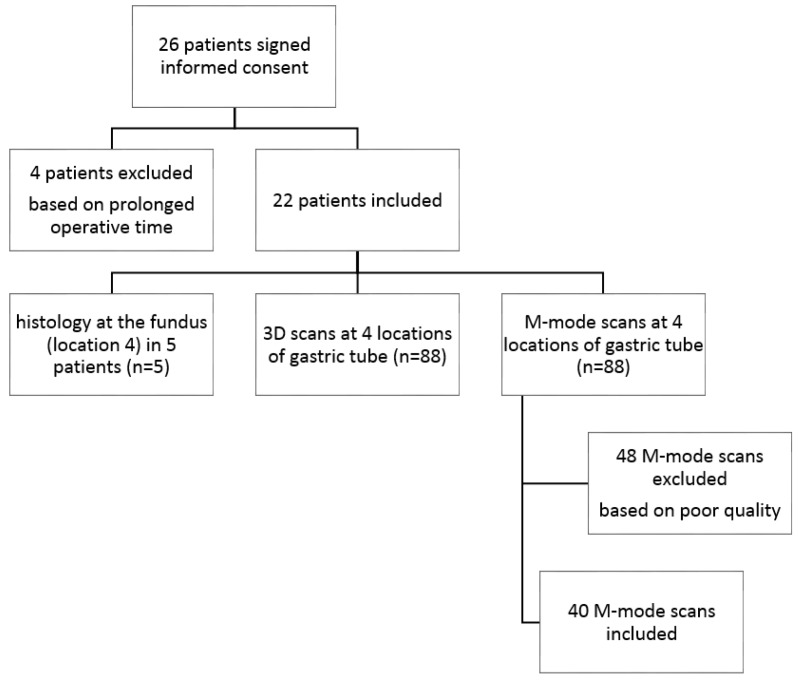
Flow diagram: patient and data inclusion.

In total, 26 patients signed informed consent. Four patients were excluded based on delay in operation time (measurements interrupted the operation by ±20 min), which made imaging impossible considering patient safety. Therefore 22 patients were included for data acquisition (Figure 4). In all 22 patients, 3D OCT images were acquired at four locations of the gastric tube, from the base to the fundus, the future anastomotic site (*n* = 88). Furthermore, M-mode scans were acquired at the same four locations of the gastric tube, from physiologically expected normal to decreased perfusion (*n* = 88). Speckle contrast analysis was not possible for all acquired M-mode scans due to poor quality of the scans (e.g., specular reflections, out of focus, and crossing zero-delay). In most cases the quality of the scans was hampered by specular reflections from the sterile sheet on top of the tissue. In total 48 M-mode scans were excluded. In four patients (*n* = 12) OCT data acquisition and speckle contrast analysis yielding areas indicative of flow (%) was successful at all four locations.

### 3.2. 3D OCT Scans

**Figure 5:**
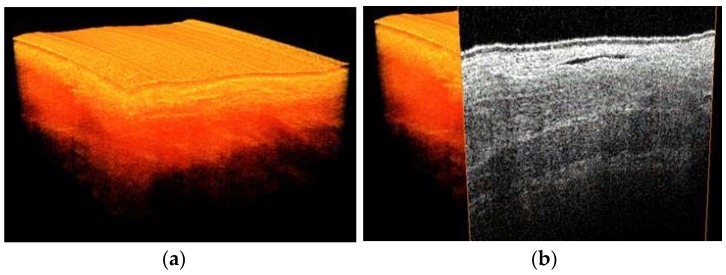
3D OCT scan of the gastric tube, (**a**) volumetric representation (**b**) with cross section visualized.

**Figure 6:**
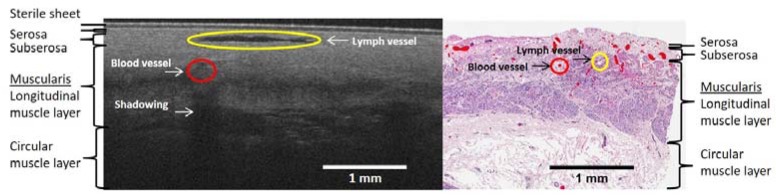
OCT B-scan of location 4, the fundus (at the left panel), and HE stained histology slide (the right panel) obtained at the end of the gastric tube. Blood and lymph vessels are indicated in red and yellow, respectively. The corresponding tissue layers, serosa, subserosa (purple/dark pink) and muscularis propria (light pink) are depicted in both panels. The scale bar depicts a length of 1 mm.

3D images were obtained at four locations of the gastric tube (Figure 5). On the 3D scans, different tissue layers could be distinguished. By comparison with histopathology slides, these layers could be identified. Importantly, the localization of the blood vessels of the reconstructed gastric tube was similar in OCT images compared to histopathology (Figure 6). Furthermore, because lymph fluid is a low-scattering medium, lymph vessels could be identified in the OCT images as well. 

### 3.3. Speckle Contrast Analysis of M-Mode OCT Scans

**Figure 7:**
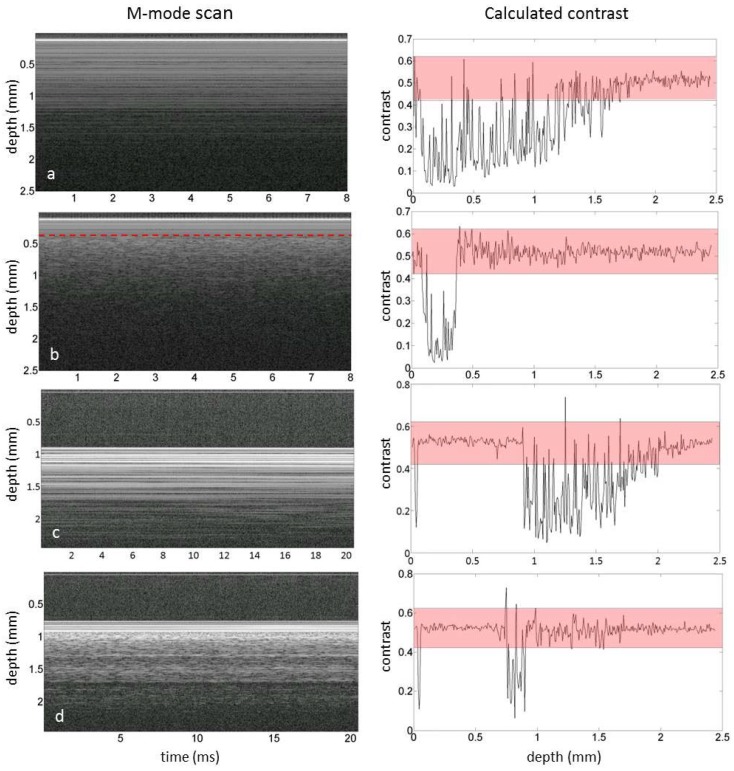
Single M-mode OCT scans (**left**) and the corresponding calculated contrast (**right**) of a. a static region of the flow phantom, b. a region with flow in the flow phantom in which the red line depicts the approximated location of the top of the flow channel, c. a static region of tissue and d. a region with flow in tissue by a blood vessel at approximately 0.2 mm below the tissue surface. The red bar depicts the speckle contrast threshold used in this manuscript.

Areas indicative of flow could be distinguished from static tissue by calculating the speckle contrast in the M-mode images. As depicted in Figure 7A, the contrast calculated for a single M-mode scan for static tissue was mostly below 0.4, unless the SNR was too low as observed at larger depths. When flow was present in the channel at 0.2 mm below the surface, the speckle contrast was between 0.42 and 0.62 (Figure 7B). Please note that an increase in the speckle contrast was observed below the flow channel as well. Similar effects were observed in the single M-mode scans of the reconstructed gastric tube, as depicted in Figure 7C for the less perfused part of the tissue and in Figure 7D in which a blood vessel was present at a depth of 0.2 mm below the tissue surface. 

Calculation of speckle contrast percentage (%) as a parameter for tissue areas with flow was possible in 13 (*n* = 40) of the 22 included patients (59%) and in all four locations in 4 (*n* = 12) of the 22 patients (14%) (for data inclusion and exclusion criteria, see first results section and Figure 4). We observed a decrease in speckle contrast percentage (%) from location 1 to location 4 in 6 of the 13 patients (46%) and an increase in 3 of the 13 patients (23%). The speckle contrast percentage data at location 1 or 4 was missing for 3 out of 13 patients.

**Figure 8:**
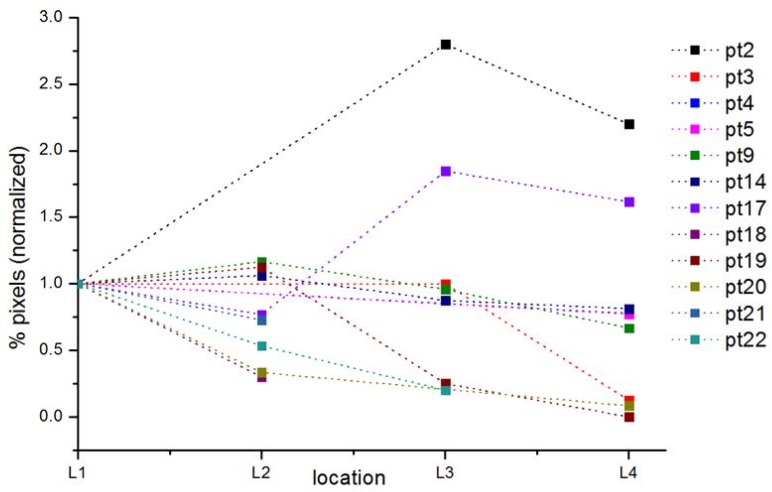
Normalized percentage of pixels per patient per location obtained from the OCT M-mode scans. The values are normalized relative to location 1, hence only the plots for patients with a value for location 1 are shown.

The results, percentage pixels indicating flow relative to the total number of pixels, per patient and location are summarized in Table 1 and Figure 8. In 10 patients, data analysis was possible for location 4. In 80% of these patients, percentage of flow pixels was lower compared to location 1, 2 or 3. 

**Figure 9:**
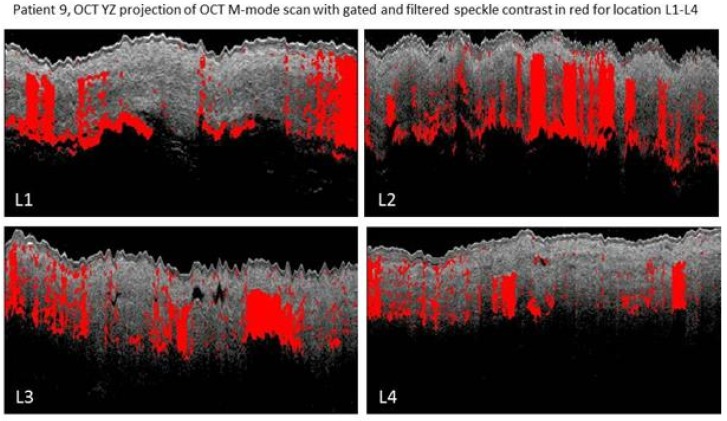

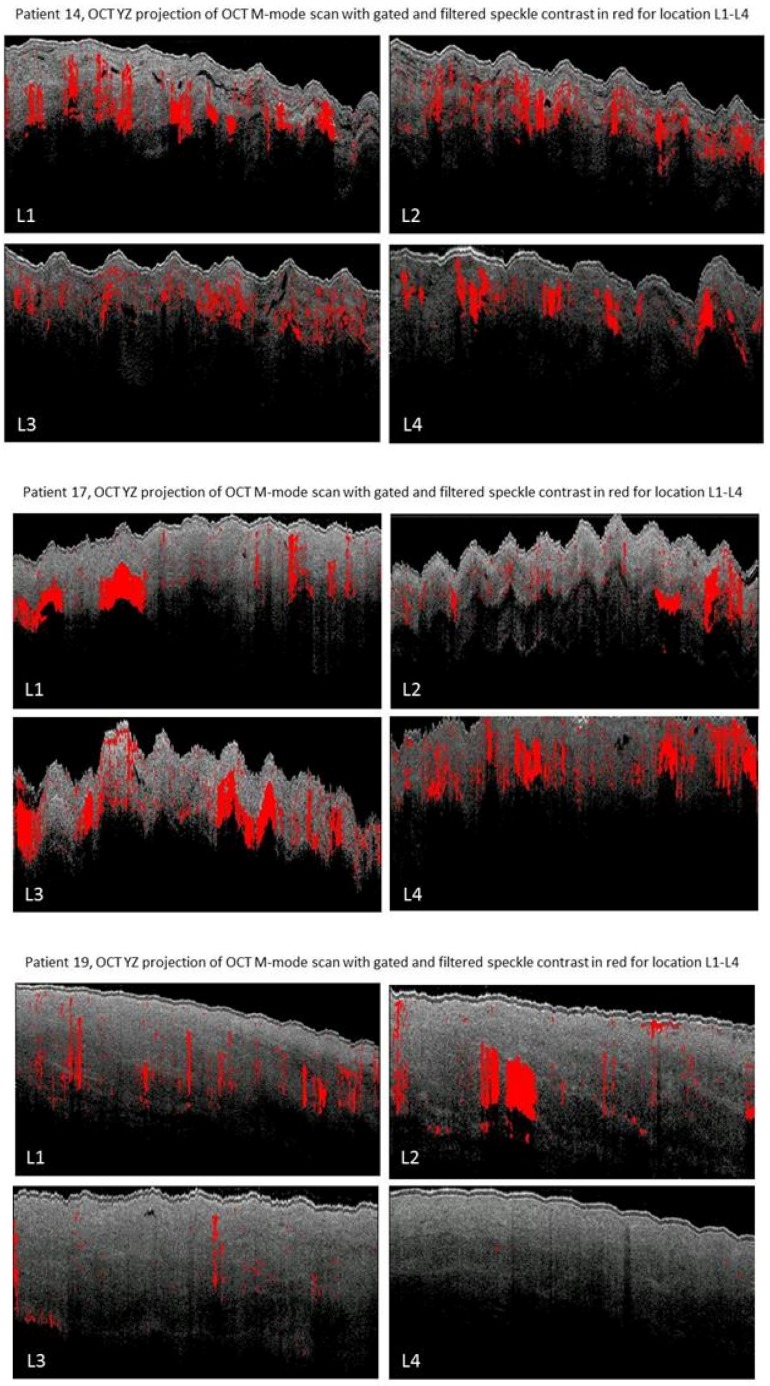
OCT speckle contrast indicative of flow overlaid with OCT grayscale (YZ) images from M-mode scans from location 1 (L1, 3 cm below the watershed), location 2 (L2, watershed), location 3 (L3, 3 cm above the watershed), location 4 (L4, fundus). In patient 9 high speckle contrast indicative of flow is seen in location 2 on the watershed area. In patient 14 and 19 a decreased speckle contrast indicative of flow is observed towards location 4. In patient 17 an increase in speckle contrast indicative of flow from location 1 to 4 is observed.

For patients 9, 14, 17 and 19 the speckle contrast analysis was possible on all four locations on the gastric tube. Figure 9 depicts the OCT M-mode scans of all four locations for patients 9, 14 17 and 19. A decrease of areas with speckle contrast indicative of flow (red) is visible towards the fundus in patients 9, 14 and 19. The expected shadowing due to multiple scattering [8] caused by the high scattering coefficient in blood is clearly observed in the images [18].

### 3.4. Histology Results

**Figure 10:**
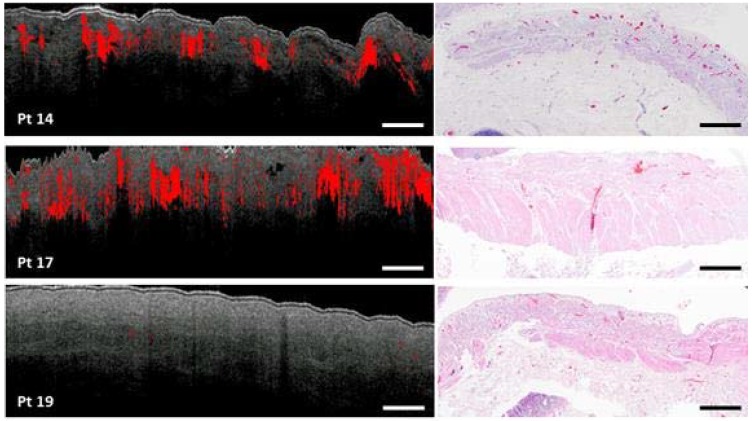
OCT scan of location 4 (fundus), the scale bar depicts a length of 1 mm, and histology slides HE-stained, of patient 14, 17 and 19 with blood vessels in red and tissue layers: serosa, subserosa (purple/dark pink), muscularis propria (light pink).

Histology of the fundus tissue was available for patients 14, 17 and 19. Figure 10 depicts the OCT M-mode scan of location 4 with speckle contrast in red and the histopathology slide also of location 4 with blood vessels in red and serosa, subserosa (purple/darkpink) and muscularis propria (light pink) tissue layers. 

Although the OCT scan and the histology slide are not one to one correlated, the amount and location of the blood vessels tend to agree per patient. Histology of patient 14 demonstrates many blood vessels localized in the superficial subserosa, which is evidently visible in the OCT scan. Histology of patient 19, in contrary, shows no blood vessels, except for capillaries, which is demonstrated in the OCT scan as well. 

## 4. Discussion

This study is the first that demonstrates OCT imaging of gastric tissue and detection of flow in vivo in patients with esophageal cancer during surgery. We show that intra-operative OCT imaging of gastric tissue and microcirculation is feasible. Moreover, regions of flow could be distinguished from static tissue by calculating speckle contrast in the M-mode scans. The percentage of pixels distinguished as flow was quantified as an objective parameter. This parameter can potentially be used to differentiate normal from decreased perfusion areas. 

By comparing OCT data with HE-stained histopathology slides, it was possible to define tissue layers (serosal, subserosal, muscularis propria) and blood vessels. A network of blood vessels was observed in the subserosa. Similar blood vessels, in turn, were depicted in the OCT data exhibiting speckle contrast values between 0.42 and 0.62. Together, these findings substantiate our hypothesis that OCT speckle contrast in M-mode scans can be used to indicate regions of blood flow, while the tissue under study is moving due to the heart beat and respiration. 

Lymph vessels were visible as well in the OCT images, assuming that the lymph fluid is a low-scattering medium [19]. Detection and segmentation of the lymphatic vessel, which is outside our scope in the presented work, could potentially add to the analysis of the reconstructed gastric tube OCT data. The clinical value of visualization of lymphatic vessels in the reconstructed gastric tube has yet to be studied.

A limitation of this study is the small number of patients with successful data analysis at all locations. Quality of the images was suboptimal as stabilization of the OCT probe was very difficult. Due to the intra-operative setting, a sterile operational field and hence a sterile drape over the OCT probe was required. This sterile drape introduced specular reflections, which hampered the automated image analysis. This problem could be solved by introducing a sterile probe. Mechanical stability is a general requirement for successful OCT data acquisition and particularly for the quantification of speckle-related parameters. Motion artefacts induced by the surgeon’s hand as well as the patient’s heartbeat and breathing diminished the quality of the OCT scans by introducing non-flow related speckle decorrelation. Next to visual information, we attempted to visualize blood vessel in the 3D OCT by calculating the speckle decorrelation in adjacent B-scans using the algorithm proposed by Gong et al. [12]. Unfortunately, due to external motion artefacts we were not able to visualize flow in these scans as the speckle in most parts of the scan (also the static tissue) was decorrelated. Previous literature showed better results of microvascular OCT imaging using fixation of the probe [20], which in this study was impossible due to the in vivo, intra-operative setting of this study. Moreover, heartbeat and breathing of the patient will be a problem in imaging intestinal organs, compared to extremities, since the organs are highly perfused and therefore connected to arteries with a large diameter. For future studies, we recommend a probe stabilizer with negative pressure, as is used in SDF imaging, to decrease motion artefacts [21]. Furthermore, optimization of the scanning protocol could increase feasibility of OCT imaging: by using a smaller scanning range, heartbeat and breathing will have less influence on the motions artefacts. Equally, a faster OCT system would decrease the influence of motion in the image and increase speckle stability. 

OCT provides depth resolved images with a microscale resolution potentially allowing for visualization of the microvasculature. The visualization of blood vessels located directly underneath other blood vessels is hampered by the shadowing effect caused by multiply scattered photons in the vessels affecting both the OCT intensity and the speckle decorrelation. Red blood cells are highly forward scattering at common OCT wavelengths, which increases the probability of multiple scattering and therefore shadowing. This effect is clearly visible in Figure 7b, in which flow induces the speckle contrast of lower static parts to increase to values similarly to those of flowing blood.

The advantage of OCT over other optical modalities is the depth resolution provided in real-time. Previous research shows the potential benefit of different optical techniques in intra-operative perfusion imaging such as fluorescence imaging [4,22,23,24,25,26], thermography [27], laser speckle contrast imaging [28] and sidestream darkfield microscopy [8,29,30]. Fluorescence imaging creates a wide field overview of the vasculature of the tissue, enabling the surgeon to indicate the perfusion status of an organ or tissue by the intensity measurements of a fluorophore (e.g., indocyanine green, ICG). However, overlaying vessels cannot be distinguished. Therefore, microvascular tissue with impaired perfusion could look highly perfused because of the high flow in an underlying artery. Moreover, for the illumination of vessels with fluorescence imaging, a fluorophore is needed which makes this technique invasive. Thermography and laser speckle contrast imaging are both widely tested in vivo in patients. They both create an overview of the tissue perfusion in a color-coded scale, easily interpreted by clinicians. The disadvantage of thermography for intraoperative perfusion monitoring is the used parameter: temperature, is a parameter exhibiting a slow response to a change in tissue perfusion. Laser speckle contrast imaging, on the other hand, uses perfusion units to estimate the perfusion status, which is an arbitrary unit and therefore not easily interpreted as an absolute value to differentiate good from decreased perfusion. Sidestream darkfield microscopy provides tissue imaging, like OCT, on a millimeter scale. It is able to visualize single erythrocytes flowing through capillaries. However, it can only focus at one imaging depth up to 500 micrometer and surgeons need to focus the camera by hand, which is a challenge considering the motion artefacts discussed previously. 

OCT could optimize surgery by improving the understanding of perfusion and intra-operative visualization of the microcirculation. Integration of the software is needed to create real-time evaluation of perfusion in tissue. The proposed speckle analysis algorithm is fast, automated and can be used unsupervised, and can be integrated in the image acquisition software to enable real-time visualization of blood flow during surgery. With this parameter, real-time intra-operative OCT data analysis will be possible. Future studies should focus on the speckle contrast percentage indicative of flow and patient outcome to study a possible correlation and define a threshold value to help the surgeon to decide whether to adjust the surgical plan or not. In the future, OCT-based quantitative perfusion imaging and -evaluation could potentially improve surgical outcome and decrease post-operative complications due to impaired perfusion.

## 5. Conclusions

This study shows the feasibility of intra-operative OCT-based imaging of gastric tissue and detection of flow in blood vessels in patients with esophageal cancer. Flow was detected by calculating off-line the speckle contrast in M-mode OCT images, from which the percentage of pixels indicative of flow was obtained. This objective parameter was obtained while the bulk tissue was moving and therefor it may be a useful for intra-operative perfusion evaluation. Potentially, surgeons could use a threshold value for quantitative assessment of the perfusion state of tissue and with that improve patient outcome. 

## Figures and Tables

**Table 1:** Percentage of pixels indicative of flow per patient per location obtained from the OCT M-mode scans. In red the flow in location 4 of the gastric tube.

Patient	pt2	pt3	pt4	pt5	pt6	pt9	pt12	pt14	pt16	pt17	pt18	pt19	pt20	pt21	pt22
location 1	5	8	18	22	x	24	x	16	x	13	20	8	12	11	15
location 2	x	x	x	x	x	28	2	17	8	10	6	9	4	8	8
location 3	14	8	x	x	x	23	5	14	x	24	x	2	x	x	3
location 4	11	1	14	17	18	16	x	13	x	21	x	0	1	x	x
